# Spatial Patterns in Hospital-Acquired Infections in Portugal (2014–2017)

**DOI:** 10.3390/ijerph18094703

**Published:** 2021-04-28

**Authors:** Hugo Teixeira, Alberto Freitas, António Sarmento, Paulo Nossa, Hernâni Gonçalves, Maria de Fátima Pina

**Affiliations:** 1 MEDCIDS—Department of Community Medicine, Information and Health Decision Sciences, Faculty of Medicine, University of Porto, 4200-319 Porto, Portugal; alberto@med.up.pt (A.F.); hernanigoncalves@med.up.pt (H.G.); 2 CINTESIS—Center for Health Technology and Services Research, Faculty of Medicine, University of Porto, 4200-450 Porto, Portugal; 3 INEB—Instituto de Engenharia Biomédica, Universidade do Porto, 4200-135 Porto, Portugal; asarment@med.up.pt (A.S.); fpina@ineb.up.pt (M.d.F.P.); 4 i3S—Instituto de Investigação e Inovação em Saúde, Universidade do Porto, 4200-135 Porto, Portugal; 5 Department of Infectious Diseases, Centro Hospitalar Universitário de São João, Alameda Professor Hernâni Monteiro, 4200-319 Porto, Portugal; 6 CEGOT, Centre of Studies in Geography and Spatial Planning, University of Coimbra, 3004-530 Coimbra, Portugal; paulonnossa@gmail.com; 7 Department of Geography and Tourism, University of Coimbra, 3004-530 Coimbra, Portugal; 8 ICICT/FIOCRUZ, Instituto de Comunicação e Informação Científica e Tecnológica em Saúde/Fundação Oswaldo Cruz, 21040-900 Rio De Janeiro, Brazil

**Keywords:** hospital-acquired infections, spatial epidemiology, age-standardized hospitalization rates, spatial autocorrelation, Portugal

## Abstract

Background: Hospital-Acquired Infections (HAIs) represent the most frequent adverse event associated with healthcare delivery and result in prolonged hospital stays and deaths worldwide. Aim: To analyze the spatial patterns of HAI incidence from 2014 to 2017 in Portugal. Methods: Data from the Portuguese Discharge Hospital Register were used. We selected episodes of patients with no infection on admission and with any of the following HAI diagnoses: catheter-related bloodstream infections, intestinal infections by *Clostridium difficile*, nosocomial pneumonia, surgical site infections, and urinary tract infections. We calculated age-standardized hospitalization rates (ASHR) by place of patient residence. We used empirical Bayes estimators to smooth the ASHR. The Moran Index and Local Index of Spatial Autocorrelation (LISA) were calculated to identify spatial clusters. Results: A total of 318,218 HAIs were registered, with men accounting for 49.8% cases. The median length of stay (LOS) was 9.0 days, and 15.7% of patients died during the hospitalization. The peak of HAIs (*n* = 81,690) occurred in 2015, representing 9.4% of the total hospital admissions. Substantial spatial inequalities were observed, with the center region presenting three times the ASHR of the north. A slight decrease in ASHR was observed after 2015. Pneumonia was the most frequent HAI in all age groups. Conclusion: The incidence of HAI is not randomly distributed in the space; clusters of high risk in the central region were seen over the entire study period. These findings may be useful to support healthcare policymakers and to promote a revision of infection control policies, providing insights for improved implementation.

## 1. Introduction

A nosocomial infection, also known as hospital-acquired infection (HAI), is defined as an infection acquired by a patient while receiving health care [1,2]. These infections are usually developed during hospitalization and manifest no earlier than 48 h after the hospital admission or up to 30 days after receiving health care, in the case of Surgical Site Infections (SSI) [3]. Despite HAIs being more frequently identified in hospitalized inpatients, they also include infections detected after discharge or occupational infections among the health staff [4]. Usually, HAIs are caused by bacterial, viral, or fungal pathogens, where the most common types include the bloodstream infections related to the central venous catheter [5,6,7], hospital-acquired pneumonia [8,9], intestinal infections by *Clostridium difficile* [10,11], the SSI [12,13], and urinary tract infections associated with catheter use [14,15].

Hospital-acquired infections represent one of the most frequent adverse events during healthcare delivery; they may result in prolonged hospital stays, long-term disability, increased resistance to antimicrobials, or even death [16,17]. Despite their relevance, the real burden of HAIs remains unknown due to the complexity of the various surveillance systems and the lack of uniform diagnosis criteria from country to country [18]. According to several studies, the prevalence of HAIs in middle-income countries ranges from 5.7% to 19.1% of all hospitalizations, whereas in developed countries, it varies between 5.7% and 7.5% [19,20,21,22]. However, in some high-income countries, the prevalence has been reported to be as high as 12.0% [23]. According to data provided by the Portuguese Directorate-General of Health in the scope of the report of the 2nd European survey on the prevention program for infection control in 2017, the prevalence of HAIs in Portugal was around 7.8%, showing a decreasing trend since 2012 [24,25]. Nonetheless, the literature indicates, for the Portuguese case, an absence of epidemiological data on HAIs in individual Portuguese Intensive Care Units (ICUs), which makes it difficult to compare data and impairs the understanding of any spatial differences in prevalence, which may be associated with the area of influence of the ICUs, according to the national referral network [26].

Moreover, HAIs are directly or indirectly responsible yearly for more than 148,000 deaths in Europe and around 98,000 in the USA [2,27,28]. Despite these numbers, hospitals take hospital-acquired infections very seriously. To reduce the impact of these infections, several hospitals worldwide have implemented infection tracking and surveillance systems as well as solid prevention strategies [29]. Prevention and minimization of HAIs are the responsibility of all health actors and include infection control programs, infection control committees, and staff training [30,31].

Regarding surveillance, several hospitals have used data from health information systems, such as hospital discharge registers, as an automated alternative instrument to complement diagnosis and to improve process efficiency and precision [32,33,34]. Although the accuracy of administrative coded data is affected by coding process subjectivity and the variability of distinct coding versions [35,36,37,38], the information based on this type of data is internationally recognized [34,39]. Its use has been successfully applied for several research purposes, including to assess some HAIs [39,40,41].

The use of hospital discharge data for the assessment and description of HAIs as well as inference about these infections can give important clues about their trends and patterns. To our knowledge, no studies have provided a descriptive analysis of HAIs in Portugal or in other European countries using administrative data of hospitalizations. Our study aims to fill this gap, describing the spatial patterns of the Age Standardized Hospitalization Rates (ASHRs) of HAIs and exploring the existence of spatial clusters in mainland Portugal from 1 January 2014 to 31 December 2017.

## 2. Materials and Methods

### 2.1. Study Area

The study area is mainland Portugal, located on the Iberian Peninsula in southwestern Europe, with a land area of 89,102 km^2^. The mainland was estimated to have 9,792,797 inhabitants in 2017 (43.0% aged 50 and over), distributed heterogeneously throughout five regions and 278 municipalities (with a population varying from 1634 to 509,515 inhabitants). The North, Center, and Lisboa Regions held 88.8% of the Portuguese population in 2017, while Alentejo (7.3%) and Algarve (4.5%) had much lower proportions.

Portugal is a developed country, despite presenting a high GINI index value (32.1 in 2017), meaning that the inequality of wealth distribution is one of the highest in the European region [42].

The national health system is universal, allowing global coverage to all residents despite their socioeconomic, legal, or employment status. It contains three coexisting systems: the national health service, the health subsystems for specific professions, and the private health sector [43].

### 2.2. Study Design

We carried out a retrospective and observational population-based ecological study using secondary data from public hospital admissions. The respective geographic and temporal units of analysis were the municipality and the calendar year for the period of 1 January 2014 to 31 December 2017.

### 2.3. Data Sources

We obtained hospitalization data from the Portuguese Hospital Discharge Register, managed by the Central Administration of the Health System (ACSS) of the Portuguese Ministry of Health. These data refer to hospital admissions in public hospitals and are provided for research upon request. Each record corresponds to one hospital admission and contains the following information: sex (male or female); age and date of birth; municipality of the patient’s residence; external causes of injury, coded according to the International Classification of Diseases (ICD); principal diagnosis (and secondary diagnoses) coded according to the ICD; medical or surgical interventions (also represented with ICD codes); type of admission (unplanned admissions—admissions through the emergency department); dichotomy (yes/no) indicator of infection Present On Admission (POA) indicator; which hospital is providing the care; outcome (for example, discharge home, discharge to another hospital, deceased); Length of Stay (LOS); geographic units of the patient’s place of residence; and Diagnosis Related Groups variables. Registers were coded according to ICD version 9, Clinical Modification (ICD-9-CM) for the years 2014 and 2015 as well as a significant part of 2016, and ICD version 10-CM was used for the remaining period. More detailed information about the variables used can be found in Table S1. In the studied period, no such data were available for the Portuguese archipelagos of the Azores and Madeira, and therefore they were not included in this study.

For the study period, population estimates were obtained from the National Institute of Statistics (INE) [44], which were aggregated by municipality, sex, and 5-year age groups.

### 2.4. Data Selection

Given the nature of the studied condition and the accuracy of the coding systems used, a review analysis was conducted, using the best available scientific evidence [33,39,40], to obtain a consensual list of codes to characterize the most common HAI contexts. The codes were organized by context and validated through discussions with physicians and specialists in the fields of medical coding and infection control, considering both coding systems. Table 1 shows the selected diagnoses in our analysis.

We selected all in-patient episodes with a discharge date between 1 January 2014 (first year with available POA indicator) and 31 December 2017 (latest available and validated data) from the Portuguese Hospital Discharge Register with any HAI suggested diagnosis through the ICD code, combined with a negative POA indicator.

Each hospitalization was considered an independent episode. We excluded episodes with a LOS of less than three days to conform with the HAI definition. Episodes with more than 180 days (*n* = 246, 0.07%) were excluded due to prolonged hospitalizations that may be associated with lack of social support (e.g., older adults without a place in a nursing home).

The Charlson Comorbidity Index (CCI) was calculated through the identification of specific comorbidities using secondary diagnosis. The CCI categorizes comorbidities of patients based on the ICD diagnosis codes [45,46]. A weight is assigned to each comorbidity group based on resource use and adjusted mortality risk. The index score results in the sum of all weights. A score of zero means that no comorbidities were found, while a higher score indicates a higher chance of developing a weak general health status, which would require the consumption of more resources [47]. More detailed information can be found at Freitas et al. [48].

### 2.5. Data Analysis

Geographic Information Systems (GISs) and spatial statistical techniques were used to analyze the data. Due to the high differences observed in HAI incidence by age groups, data were analyzed globally and according to the following categories: youth (0–19 years), adults (20–64 years), and elderly (65 or more years). Descriptive statistics, such as the median (interquartile range) for the quantitative variables and the absolute (relative) frequencies for the categorical variables, were calculated for each sociodemographic and clinical characteristic using IBM SPSS Statistics 26 for Windows (IBM Corp., Armonk, NY, USA).

The age-standardized hospitalization rates (ASHRs) of HAIs, per municipality and year, were calculated using the direct method, with the European population as standard [49] and five-year age groups (from 0 to 100 or more). To overcome the statistical instability caused by the Problem of Small Numbers [50] in municipalities with a small population, we used the empirical Bayes (EB) method (Equation (1)) [51] to smooth the local risk. This approach is a statistical estimation based on the observed data, where the degree of “smoothing” is calculated according to a weight that varies from 0 to 1 as a function of the population size and the variability of the ASHR in the neighborhood. Therefore, for municipalities with large populations and thus not affected by the statistic instability, the weight is close to 1, meaning that the adjusted rates are like the observed rates. On the other hand, the lower the weight, the less we “trust” in the observed rates (because they can be artificially high due to the Small Number Problem); therefore, they are smoothed to the average of the neighbors [52].

(1) $$\mathrm{EB}\;\mathrm{ASHRi}=\left(\mathrm{ASHRi}\times Wi\right)+\left({\mathrm{ASHR}}_{\mathrm{neighorhood}}\times \left(1-Wi\right)\right)$$

$$\begin{array}{l} \mathrm{ASHRi}=\mathrm{age}\;\mathrm{standardized}\;\mathrm{hospitalization}\;\mathrm{rate}\;\mathrm{in}\;\mathrm{the}\;\mathrm{municipality}\;i\\ Wi=\mathrm{weight}\;\mathrm{in}\;\mathrm{municipality}\;i\\ {\mathrm{ASHR}\;}_{\mathrm{neighorhood}}=\mathrm{neighborhood}\;\mathrm{age}\;\mathrm{standardized}\;\mathrm{hospitalization}\;\mathrm{rate} \end{array}$$

We used first order neighborhoods calculated using the “queen contiguity” method, which considers as neighbors all the municipalities that share at least one vertex. After defining the neighbors of each municipality, we summed the cases and population of each neighbor and calculated the ${\mathrm{ASHR}}_{\mathrm{neighorhood}}$ using the direct method and European standard population as described before. In summary, the estimated EB ASHR better describes the risk in a municipality by smoothing the artificially high observed ASHR caused by few cases in a small population.

Using the EB ASHR, we calculated the Moran’s Index according to Equation (2) [53], to measure the presence of spatial autocorrelation. The Moran index is a global indicator of autocorrelation, where a score close to zero means that there is no autocorrelation, with events arising randomly in space. A score near −1 or 1 represents a strong (negative or positive) autocorrelation, implying a spatial dependency in the event occurrence. However, when dealing with many areas, different local spatial associations may occur. Therefore, we also computed the Local Index of Spatial Autocorrelation (LISA) using Equation (3) [54].

(2) $$I=\frac{n\sum w_{ij}\;\left(z_{i}-\bar{z}\right)\left(z_{j}-\bar{z}\right)}{S_{o}\sum_{i}{\left(z_{i}-\bar{z}\right)}^{2}}$$

(3) $$I_{i}=\frac{z_{i}\sum_{j=1}^{n}w_{ij}z_{j}}{\sum_{j=1}^{n}z_{j}^{2}}$$

where *n* = number of areas; $z_{i\;}$= value of the variable considered in area; $\bar{z}$ = the variable’s average value in the study area; $w_{ij}$ = elements of a well-balanced matrix, based on spatial proximity; $z_{j}$ = variable value of the considered *j* area.

The LISA identifies areas where the ASHR is significantly correlated with the ASHR of their neighbors [55]. Based on LISA results, four types of clusters were identified: high-high (areas of high ASHR, with neighbors also with high ASHR), high-low (areas with high ASHR surrounded by areas with low ASHR), low-high (areas with low ASHR surrounded by areas with high ASHR) and low-low (areas of low ASHR, with neighbors also with low ASHR). The GeoDa 1.16.0.12 software (University of Chicago, Chicago, IL, USA) was used to calculate the Moran Index and LISA, and ArcGIS 10.5.1 (ESRI, Redlands, CA, USA) was used to map the results.

### 2.6. Ethics Statement

The secondary data from the Portuguese Hospital Discharge Register was obtained following the current Portuguese legislation. The availability of these anonymized data does not require specific approval from ethical committees. The global research was approved since it did not include samples or experiments on humans or their personal information.

## 3. Results

During the study period, and according to the selected criteria, there were 320,288 episodes of hospitalizations with one or more HAIs. Of the total, 2070 episodes (0.65%) were disregarded because of missing information related to the patient residence municipality, leaving 318, 218 episodes of the analyzed population. Median (Interquartile range—IQ) age was 77.0 years (20.0) for men and 81.0 years (17.0) for women.

### 3.1. Profiles of HAI Cases and Their Sociodemographic and Clinical Characteristics

**Figure 1:**
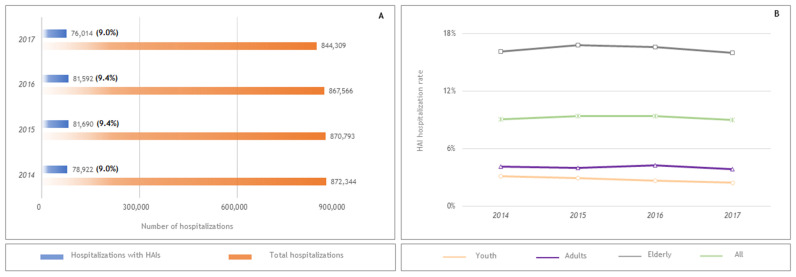
(**A**) Yearly number of hospitalizations with Hospital Acquired Infections and the total number of hospital admissions. (**B**) HAI hospitalization rate per age category.

The yearly average number of episodes of HAI was 79,555, corresponding to approximately 1525 cases per week. The yearly number of hospitalizations with HAI exhibited an increase from 2014 to a peak in 2015 (*n* = 81,690), representing 9.4% of the hospital admissions, followed by a decrease until a global minimum in 2017, with an HAI incidence of 90.0 cases per 1000 admissions (Figure 1).

This pattern was also observed within each age category. Within the whole study period, youth (0–19 years) accounted for 4.7%, whereas adults (19–64 years) and elderly (65 or more years) accounted for 18.1% and 77.2%, respectively. Regarding the distribution of cases by sex, men accounted for 49.8%. The age range was 0–109 years, with a median (IQ) age of 79.0 (20.0). The median length of stay was 6.0 (5.0) days for the youth and 10.0 days (10.0) for the elderly; 94.3% of patients were admitted urgently, while 5.7% were admitted in a scheduled way (Table 2).

There were 50,087 patients (15.7%) who died during their stay, while the majority (78.0%) were discharged home. The elderly age category had the highest percentage of deaths (18.4%) during their hospital admission, while the youth presented the lowest, with 0.7%.

The North region, with a higher concentration of inhabitants, had a higher frequency of HAIs, with 100,933 (31.7%) cases. All age groups reflect these general values, except for youth in the Lisboa region, where the highest frequency was registered (36.5%) when compared with the others.

There were differences in the Charlson comorbidity index (CCI) for the HAI inpatients between the different age groups. Most of the youth (87.1%) did not have any pre-existing conditions, while in the opposite direction, 60.6% of the adults and 81.8% of the elderly registered at least one or more comorbidities.

Nosocomial pneumonia was the most common HAI in all age categories, with 197,188 hospitalizations (58.0%); urinary tract infections were the second most common, with 107,651 hospitalizations (31.7%). Overall, intestinal infection by *Clostridium difficile* was the least frequent, with 3822 (1.1%) episodes.

A minority of in-hospital deaths in patients with an infection acquired after surgery was verified (5.0%). Unsurprisingly, the lethality was higher for patients with nosocomial pneumonia, with 18.5% deceased during their hospital admission.

Although admissions of patients with intestinal infection by *Clostridium difficile* represented the least frequent event (1.1%), the data showed that 16.6% presented a fatal outcome (Table 3). In summary, almost all HAI contexts present a lethality above 10%.

The surgical site infection context was the most frequent (54.3%) among patients without comorbidities (Table 4), whereas urinary tract infections were the most common among patients with at least one comorbidity (77.7%). On the other hand, catheter-related bloodstream infections were the most frequent in patients with three or more pre-existing pathologies (36.9%).

### 3.2. Spatial Distribution of Hospitalization Rates by Municipality

**Figure 2:**
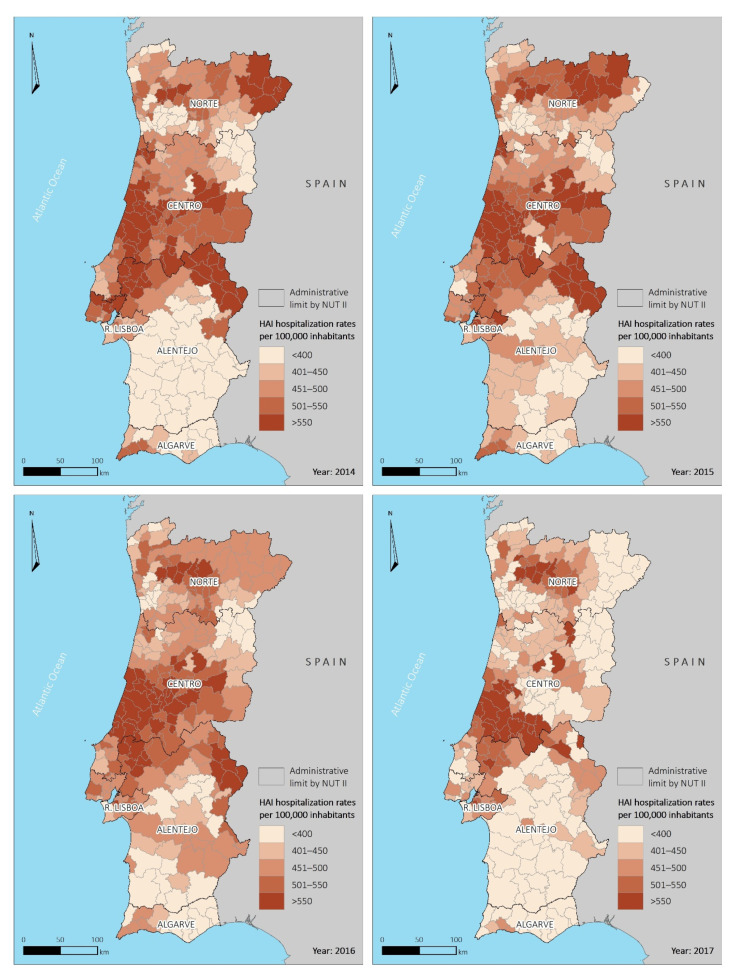
Spatial distribution of age-standardized HAI hospitalization rates per 100,000 inhabitants, per municipality, for the period 2014–2017.

The spatial distribution of the ASHRs of HAIs by 100,000 inhabitants per municipality and by year is shown in Figure 2. Substantial spatial disparities were verified, with ASHR values in the range of 256.0–846.8 episodes/100,000 inhabitants in 2014, and 306.3–1109.2 episodes/100,000 inhabitants in 2015, with the highest rates in the central region of the country and lower in the south.

Between 2014 and 2015, the ASHR increased; however, from 2015 to 2017, we observed a global decrease throughout the mainland. In particular, the mean (standard deviation) of episodes per 100,000 inhabitants was 490.8 (78.4) in 2015 and 435.5 (72.2) in 2017. Moreover, the proportion of municipalities in the two highest quintiles decreased from 41.0% in 2015 to 20.9% in 2017. Despite this reduction, during the study period, the municipalities in the north and central region had consistently higher ASHRs than those in the south.

**Figure 3:**
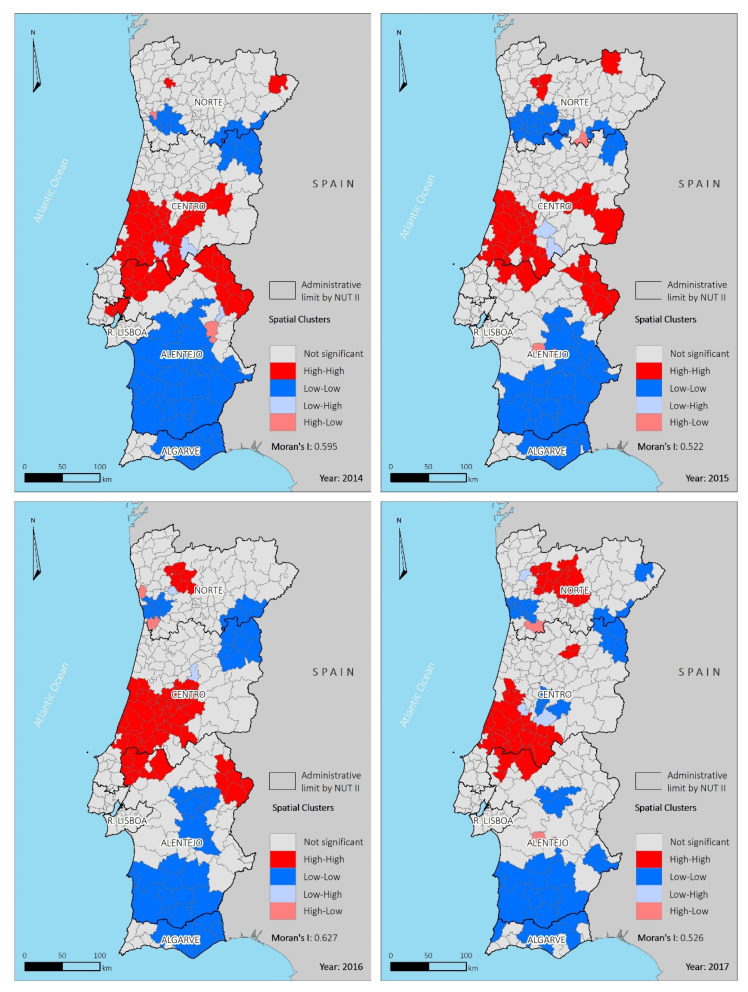
The spatial clusters of ASHR by municipality for the period 2014–2017.

The Moran index was moderate to high in the studied period, being higher in 2016, with a value of 0.627 (*p* < 0.05), meaning that the ASHR does not occur randomly in the space. Spatial clusters of high and low ASHR were identified with LISA analysis (Figure 3). The most significant high-high clusters were located mainly in the center region of the country, with some smaller high-high clusters found in the north. A total of three low-risk clusters were identified between 2014 and 2015, while between 2016 and 2017, the number of low-risk clusters increased to four and six, respectively, despite a size reduction.

The largest low-risk cluster was situated in the southern municipalities, while a smaller low-risk cluster was concentrated in the northeast. No clusters were identified in the Lisboa region during the study period. Geographic disparities remained when data were analyzed by categories of age (Figure S1), with the ASHR variability higher in the older age categories (ranging between 1281.4 and 4886.4 cases per 100,000 inhabitants). The adults had ASHR values ranging from 99.5 to 571.1 cases per 100,000 inhabitants, while the youth had values between 26.2 and 997.6. In terms of the LISA, when compared to the other two age groups (adults and the elderly), the youth had a slightly different pattern, with higher clusters in the eastern area of the country and lower clusters of cases on the coast over the four years. More detailed information about the values per year ASHR and the Moran index can be found in Supplementary Tables S2 and S3.

## 4. Discussion

To the best of our knowledge, this is the first nationwide population-based ecological descriptive study of HAIs for all age groups in mainland Portugal and in Europe. This retrospective four-year study analyzes hospital admission episodes of patients who acquired a nosocomial infection, based on data collected from a national hospital discharge register gathering information from public hospitals. The geographic distribution, the incidence, and the characteristics of hospitalized patients are described for the first time. Our findings show that the incidence of HAI is not randomly distributed in space; there are strong inequalities, with high-risk clusters remaining in the central region throughout all the study periods.

As expected, the older age category was the most vulnerable, with the results showing a higher incidence of HAIs in patients 65 years of age or older. Findings in previous studies [18,19,56] have shown that people in this age group with infection have increased morbidity and mortality than younger individuals.

The average HAI incidence by year for all age categories fluctuated between 9.0% and 9.4% during the study period, which is in line with previously published findings for developed countries [1,23,57]. The year 2015 was the worst compared with the others within the study period, registering the highest number of cases, while the year 2017 registered the lowest number of cases. The improvement during the study period could be explained by the implementation of several plans and guidelines in the Portuguese public hospitals, such as the “STOP infeção hospitalar” (STOP hospital infection) project, promoting basic infection control precautions, and improvement of epidemiological surveillance [25], reflecting the results over a more extended period.

The observed median length of stay in this study for the individuals within the older age categories was 10.0 days. According to some studies, acquiring HAI implies an average increase of 5 (18) days within the hospitalization period [58,59,60,61], meaning that if patients had not acquired the infection, they would spend fewer days in the hospital.

**Figure 4:**
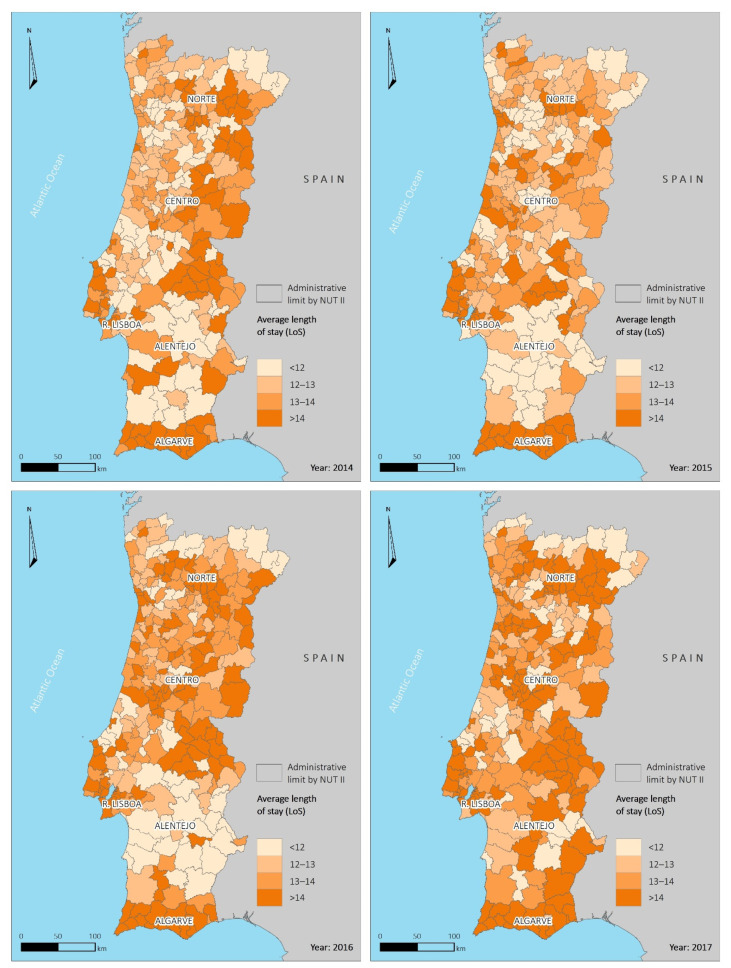
Spatial distribution of average length of stay of HAI hospitalization per municipality for the period 2014–2017.

An observation of the average hospitalization length of stay (Figure 4) for the four years, spatially distributed, shows some territorial randomization of this variable for values up to 13 days. However, we can observe some patterns of spatial distribution for lengths of stay >14 days, with more significant persistence in the southern part of the country, namely in the Algarve region, where high values are persistent over the four years, as well as in some municipalities in northern Alentejo (Portalegre district) and in some municipalities in the coastal area, located north of Lisboa. Empirically, higher values of the ageing index or longevity index could justify a longer duration of hospitalizations in the northern Alentejo area, also admitting a more remarkable number of comorbidities, but this justification is not valid for the Algarve and the coastal area north of Lisboa. It would be helpful in the future to investigate this variable broken down by hospital reference unit to check potential association patterns.

As expected, a higher CCI score was related to longer stays, possibly due to the length of time needed to evaluate and manage pre-release comorbidities and the longer time needed for recovery.

Nosocomial pneumonia was the most frequent adverse event among all HAIs, consistent with the findings reported by the Portuguese authorities [62]. Pneumonia is considered a severe problem associated with healthcare for in-patients of all ages, particularly for the youngest, who representing three quarters of the total HAI cases identified.

This may be closely linked to the use of ventilators in neonates and children, as stated in some studies [63,64,65]. Older people also have high percentages of pneumonia acquired in the hospital context, but the reasons may differ from other age categories. Several studies [66,67,68] have shown that older people have more factors of weakness, including comorbidities and other associated pathologies, which decrease their immunity and make them more vulnerable. In addition, more prolonged hospitalizations due to HAIs can consequently increase antibiotic resistance and the presence of multidrug-resistant bacteria for all patients [69]. In addition to the specificity of the demographic, clinical, and physiological characterization (comorbidity index), in the case of hospital-acquired pneumonia, the literature warns of the importance of some risk factors that, in certain situations, could be present before hospitalization: prior antibiotic treatment (previous 30 days), structural lung disease, residence in assisted living facilities/nursing homes, long-term dialysis, diabetes mellitus and immunosuppression [26,70], gastrointestinal medication (suppression of gastric acid: use of antagonists H2), and proton pump inhibitors. In addition, the poor condition of the oral cavity is a risk factor to be taken into account. In Portugal in the last six years, the high consumption of antibiotics has been the subject of awareness campaigns organized by the health authority, intending to reduce their consumption and the associated resistance. The National Program for the Prevention and Control of Infections (2017) recognized that, despite the effort to reduce the consumption of these drugs, the global consumption of antibiotics at the primary health care level remains high (21.6 daily doses per thousand inhabitants), though it is below the European average (21.9) [24]. The risk factors identified above, some of which are common to other investigated HAIs—namely urinary tract infections—indicate that medication use before a hospital episode needs to be controlled when the patient is still in the community. This constitutes a less common approach; for the most part, control recommendations fall exclusively within the hospital context, with a strong emphasis on the aseptic issues associated with clinical procedures.

### 4.1. Spatial Asymmetries

The age-standardized HAI hospitalization rates were higher in the municipalities of the central region and a few municipalities of the northern region, with some clusters within the high-risk group.

Without more detailed studies to understand the causes of such geographical patterns, it can be challenging to try to justify these patterns. It is essential to point out that this perspective of analysis does not mean that the hospital infection occurred in that region; only the municipality of residence of the patient who had been infected in the hospital environment after admission was specified, which is why we calculated the ASHR for 1000 hospitalizations as well (Figure S2). When comparing both approaches, a similar ASHR pattern distribution is shown, but the spatial clusters are considerably smaller (Figure S3). However, this cluster analysis can provide relevant insights and suggest some of the factors that could be associated with these differences, specifically for the most elderly. This includes hospitalizations for chronic diseases that would initially be preventable [71,72], reducing the number of hospital infections (since they should not be hospitalized; patients would benefit from other types of outreach care, even in a home setting, including home hospitalization).

Furthermore, the quality of the population’s access to primary healthcare and the optimization of the service performance could be determining factors [73,74,75] as it could prevent people being admitted to a hospital with greater vulnerability and a higher risk of susceptibility to infection. Another important factor is related to socio-economic and racial factors; there are already a few studies in which these factors have been found to be significant in association with patients who acquire an infection after an extended stay in hospital [76,77]. In a prospective investigation, it may be useful to cross-reference data on the consumption of antibiotics, disaggregated at the municipal scale, with the highest spatial incidence of HAIs, also controlling for the origin of the patients, specifically whether they come from their own dwelling or if they are residents in nursing homes, where the level of previous infections and antibiotic use is unknown. This approach could be beneficial for understanding the pattern of clusters found for the two main identified HAIs: nosocomial infection by pneumonia and nosocomial infection of the urinary tract. In nosocomial pneumonia, we observed the existence of high and spatially consistent values over the entire period for a wide range of municipalities located in the central region and northern Alentejo. In the extreme north of Portugal during the study period, the number of municipalities with high values decreased in the last biennium 2016–2017 (Figure S4). Something similar occurred with nosocomial urinary tract infections. During the investigation period, three clusters with high values were identified, with special emphasis on a continuous territory between the central coast and the area north of Lisboa, in the western end of the Algarve, and a set of municipalities in the interior of the Alentejo near the border. From 2016 to 2017, a cluster of high values also emerged in the northern littoral region (Figure S5).

Despite the possible clues and limitations already presented, other studies point out other reasonable justifications that may contribute to understanding some patterns, such as the total number of hospital admissions and possible variances in applying the prevention protocols applied by the hospitals [78,79,80,81,82]. This descriptive article sought to determine whether the clusters are randomly distributed in space over the period under consideration. The scope was not to determine which variables could explain the results. For that purpose, a multivariate regression model is currently under development, which will allow for the adjustment of variables and an understanding of causality.

### 4.2. Limitations

There are some limitations to this study. Specifically, due to the nature of this research, caution must be applied when analyzing data and interpreting findings from secondary sources. These results were based on information from health records summarized by medical coders, which present the possibility of bias due to possible incompleteness or inaccuracies [83]. Nevertheless, ACSS conducts regular audits on this data to ensure accuracy and quality. Another major limitation concerns the impossibility of identifying the infection cases detected after discharge within 30 days.

Even though our study methods may be applied to other countries, we cannot be certain of the replication of our results due to differences in demographics, economics, and healthcare systems.

### 4.3. Implications and Future Work

Despite its limitations, this study presents several strengths and implications. First, unlike most studies, this one looked at several HAI contexts, providing a full picture of the country’s spatial patterns of hospitalization rates, using data from patients of all ages. This result will be valuable to adjust measures and improve the action plan for the control and surveillance of nosocomial infections. Furthermore, many of the leading causes of hospitalization in Portuguese patients (e.g., diabetes mellitus) are preventable [71], and a significant portion of the population has inefficient access to primary health care. The existence of a high-risk cluster, stable in the central region of Portugal for all HAIs investigated, may be associated with an unsuitable profile of consumption of antibiotics or with a higher prevalence of patients hospitalized in nursing houses; such an evaluation is not allowed by a descriptive ecological study. Despite the efforts of the Portuguese national health system to enhance quality, inequalities in the distribution of primary care facilities remains an important issue. Many regions have low coverage of family doctors, resulting in real barriers to access and longer wait times for assistance. Official data show that Portugal has had some difficulty allocating health providers to the most rural areas [84]. As a result, improving primary health care is likely to reduce hospital admissions. Providing the necessary assistance in the development of new public health policies may be supported by targeting specific measures for the high cluster territories. Consequently, this could lead to a reduction in the pressure on hospitals, as well as a decrease in associated costs, prolonged stays, deaths, and existing morbidity [78].

Subsequent studies are warranted to understand the reasons that could be associated with these numbers and asymmetries or to explore the possibility of a hospital-related analysis within influence areas with a higher number of cases. It can be helpful to see if they have a higher burden compared to the others. Finally, developing a platform with this information to allow consultation for the regional health delegations might also benefit the country.

## 5. Conclusions

This study described the incidence of HAIs in mainland Portugal for a quadrennium. A reduction in incidence was observed between 2015 and 2017, and the most representative adverse event recorded was nosocomial pneumonia, with the elderly being the most affected. Specific regions within the country recorded higher incidences, such as the center and north, and possible justifications, such as asymmetries in access to primary health care, were discussed. As an emerging issue, it is important to promote further research, including the reorganization of healthcare systems and their guidance, the improvement of diagnosis, and the effective management of procedures.

The role of the HAI control committees within the clinical context is essential for educating health care providers, and the quality of health care must be ensured by evaluating indicators and endorsing investments with cost-effective allocation of resources. As a result, these findings can help to warn analysts in surveillance systems, leading to well-informed decisions.

## Figures and Tables

**Table 1:** ICD-9-CM/ICD-10-CM codes used to identify HAI episodes.

Hospital-Acquired Infections	ICD-9-CM Codes			ICD-10-CM Codes		
Catheter-related bloodstream infections	038.12	038.11	041.11	A41.01	A41.02	B95.61
	041.12	996.62	999.3x	B95.62	T80.2-	T82.7-
Infection by *Clostridium difficile*	008.45			A04.7-		
Nosocomial Pneumonia	480x	481	482x	A48.1	B01.2	B05.2
	483x	485	486	J10.0-	J11.0-	J12-
	487.0	997.3x		J13	J14	J15-
				J16-	J17	J18-
Surgical site infection	483x	485	486	J10.0-	J11.0-	J12-
	487.0	569.61	682x	J13	J14	J15-
	996.6x	997.3x	996.7x	J16-	J17	J18-
	998.5x	998.6	999.34	O86.0-	T81.4-	T81.8-
	999.39			T84.5	T84.6	T84.7
				T88.0-	T88.8-	Z48.8-
Urinary tract infection	590.1x	590.2	590.8x	N10	N15-	N16
	590.9	595.0	595.4	N30-	N30.81	N39.0
	599.0	996.64	997.5	N99.89	T83.5-	

**Table 2:** Sociodemographic and clinical characteristics of patients admitted in mainland Portuguese public hospitals with HAIs.

		Age Category		
Characteristics	Total	Youth	Adults	Elderly
**Total HAI hospitalizations, *n* (%)**	318,218 (100.0)	14,851 (4.7)	57,700 (18.1)	245,667 (77.2)
**Age, (years), Median, (IQR)**	79 (20.0)	2 (7.0)	54 (15.0)	82 (11.0)
**Length of stay (LoS), (days), Median, (IQR)**	9 (10.0)	6 (5.0)	10 (11.0)	10 (10.0)
**Sex, *n* (%)**				
Men	158,552 (49.8)	7921 (53.3)	33,822 (58.6)	116,809 (47.5)
Women	159,666 (50.2)	6930 (46.6)	23,878 (41.4)	128,858 (52.5)
**Charlson comorbidity index, *n* (%)**				
0	80,401 (25.3)	12,934 (87.1)	22,736 (39.4)	44,731 (18.2)
1–2	137,858 (43.3)	1751 (11.8)	21,054 (36.5)	115,053 (46.8)
3–4	63,897 (20.1)	110 (0.7)	6868 (11.9)	56,919 (23.2)
>4	36,062 (11.3)	56 (0.4)	7042 (12.2)	28,964 (11.8)
**Destination after discharge, *n* (%)**				
Residence	248,069 (78.0)	14,250 (96.0)	48,349 (83.8)	185,470 (75.5)
Hospital transfer	7484 (2.4)	421 (2.8)	2408 (4.2)	4655 (1.9)
Discharge against medical advice	881 (0.3)	23 (0.2)	505 (0.9)	353 (0.1)
Transfer to continuous care	11,697 (3.7)	58 (0.4)	1676 (2.9)	9963 (4.1)
Deceased	50,087 (15.7)	99 (0.7)	4762 (8.3)	45,226 (18.4)
**Admission type, *n* (%)**				
Scheduled	17,916 (5.6)	1280 (8.6)	6525 (11.3)	10,111 (4.1)
Unplanned	300,181 (94.4)	13,569 (91.4)	51,133 (88.6)	235,479 (95.9)
Others	121 (0.0)	2 (0.0)	42 (0.1)	77 (0.0)
**Admissions by NUT II, *n* (%)**				
North	100,933 (31.7)	4851 (32.7)	19,922 (34.5)	76,160 (31.0)
Center	87,719 (27.6)	3266 (22.0)	12,651 (21.9)	71,802 (29.2)
Lisboa Region	94,190 (29.6)	5422 (36.5)	19,768 (34.3)	69,000 (28.1)
Alentejo	22,944 (7.2)	718 (4.8)	3211 (5.6)	19,015 (7.8)
Algarve	12,432 (3.9)	594 (4.0)	2148 (3.7)	9690 (3.9)
**Hospital-acquired infections context ^1^**				
**Total, *n* (%)**	**340,125 (100.0)**	**15,074 (4.4)**	**60,608 (17.8)**	**264,443 (77.7)**
Catheter-related bloodstream infections	19,581 (5.8)	1448 (9.6)	6435 (10.5)	11,698 (4.4)
Intestinal infection by *Clostridium difficile*	3822 (1.1)	49 (0.3)	609 (1.0)	3164 (1.2)
Nosocomial pneumonia	197,188 (58.0)	10,957 (72.7)	33,064 (54.6)	153,167 (57.9)
Surgical site infection	11,883 (3.5)	522 (3.5)	5795 (9.6)	5566 (2.1)
Urinary tract infection	107,651 (31.7)	2098 (13.9)	14,705 (24.3)	90,848 (34.4)

^1^ The patient may acquire more than one type of HAI during hospitalization.

**Table 3:** Frequency of HAI hospitalizations by context, outcome (alive or deceased during the hospital stay), and in-hospital lethality rate (IL).

Hospital-Acquired Infections	Total *n* (%)	Alive *n* (%)	Deceased *n* (%)	IL (%)
Catheter-related bloodstream infections	19,581 (5.8)	16,845 (5.9)	2736 (4.9)	14.0
Infection by *Clostridium difficile*	3822 (1.1)	3186 (1.1)	636 (1.1)	16.6
Nosocomial pneumonia	197,188 (58.0)	160,762 (56.4)	36,426 (65.8)	18.5
Surgical site infection	11,883 (3.5)	11,296 (4.0)	587 (1.1)	5.0
Urinary tract infection	107,651 (31.7)	92,707 (32.6)	14,944 (27.0)	13.9

**Table 4:** Overall frequencies of HAI contexts between 2014 and 2017 by CCI classes.

Hospital-Acquired Infections	0	1–2	3–4	>4
Catheter-related bloodstream infections	5398 (27.6)	6964 (35.5)	4101 (20.9)	3128 (16.0)
Infection by *Clostridium difficile*	929 (24.3)	1592 (41.7)	823 (21.5)	478 (12.5)
Nosocomial pneumonia	47,862 (24.3)	90,414 (45.8)	38,779 (19.7)	20,133 (10.2)
Surgical site infection	6453 (54.3)	3480 (29.3)	948 (8.0)	1002 (8.4)
Urinary tract infection	24,013 (22.3)	45,178 (42.0)	24,338 (22.6)	14,122 (13.1)

## Data Availability

The data presented in this study are available on request from the corresponding author.

## Supplementary Materials

The following are available online at https://www.mdpi.com/article/10.3390/ijerph18094703/s1, Table S1: Description of the original database variables. Table S2. ASHR values (min, max, mean) per 100,000 inhabitants. Table S3. Moran index values. Figure S1: Spatial distribution of ASHR of HAI per 100,000 inhabitants, per municipality, for the period 2014–2017, by age group; A—Youth, B—Adults, C—Elderly; Figure S2: Spatial distribution of ASHR of HAI per 1000 admissions, per municipality, for the period 2014–2017; Figure S3: Spatial clusters of ASHR of HAI per 1000 admissions by municipalities for the period 2014–2017. Figure S4: Spatial clusters of nosocomial pneumonia for the period 2014–2017; Figure S5: Spatial clusters of nosocomial urinary tract infections for the period 2014–2017.
