# Supplementary material for: Spatial Patterns in Hospital-Acquired Infections in Portugal (2014–2017)
Source: Int J Environ Res Public Health. 2021 Apr 28;18(9):4703. doi: 10.3390/ijerph18094703 (PMC8124660; doi:10.3390/ijerph18094703)
Supplement: Supplementary file 1 [file ijerph-18-04703-s001.zip › New folder/Supplementary/ijerph-1166592-Supplementary 1.pdf]

Table S1: Description of the original database variables.

Variable	Description
ID	Episode identification number
Year	Civil Year, considering the discharge date.
SEX	User gender<br>1 – Male<br>2 - Female
Age	Age of the user, in years, at the date of entry.
District	User district of residence.
Municipality	User municipality of residence.
Parish	User parish of residence.
Date of admission	Date of admission of the user to the health institution.
Discharge date	User discharge date of from the health institution.
Length of stay	Total number of days spent in the health facility by the user, according to the statistical definition of length of stay.
Destination after discharge	Destination code of the user after discharge from a hospital service:<br>1 - For the home<br>2 - To another institution with internment<br>6 - Home service<br>7 - Exit against medical opinion<br>13 - Specialized aftercare (tertiary) (collected from 2011)<br>20 - Deceased<br>51 - Palliative care - medical center (collected from 2011)<br>61 - Post-hospital care (CMS 19-22, AP21) (collected from 2011)<br>63 - Long-term hospital care (CMS 19-22, AP 21) (collected from 2011)
Admission Type	Nature or method of admission of a user to a health establishment:<br>1 - Scheduled<br>2 - Urgent<br>3 - Others
Type of episode	Type of episode:<br>AMB - Indicates if the episode was performed on the Ambulatory production line<br>INT- Indicates if the episode was performed on the production line Internment
ICD version	Coding version (ICD)
ddx1	ICD-9-CM or ICD-10 code, which identifies the Main Diagnosis of the episode - the one that is considered responsible for the patient's admission to the hospital.
ddx2…ddx30	ICD-9-CM or ICD-10 Additional Diagnostics code for the episode (up to a maximum of 30).
causad1	Code of ICD-9-CM or ICD-10 of External Cause 1 that took the user to the health institution.
causad2…causad30	Additional ICD-9-CM or ICD-10 Cause Code that took the user to the health institution.
SSI	Context of hospital infection "Surgical Site"<br>0 - Absent<br>1 - Present
Clostridium_difficile	Context of hospital infection for "clostridium difficile"<br>0 - Absent<br>1 - Present
pneumonia	Context of nosocomial infection "Pneumonia"<br>0 - Absent<br>1 - Present
trato_urinario_cateter	Context of nosocomial infection "Urinary tract infection associated with the use of a catheter."<br>0 - Absent<br>1 - Present

corr_sang_cateter	Context of nosocomial infection "Bloodstream infections related to central venous catheter"<br>0 - Absent<br>1 - Present
Charlson_indexOri	Charlson's comorbidity index (CCI) score 0 (Min) - 33 (Max)
HospID_NUTSII	Identification of the location of the health institution (NUT II)<br>NORTH<br>CENTER<br>LISBON REGION<br>ALENTEJO<br>ALGARVE
