# Supplementary material for: Spatial Patterns in Hospital-Acquired Infections in Portugal (2014–2017)
Source: Int J Environ Res Public Health. 2021 Apr 28;18(9):4703. doi: 10.3390/ijerph18094703 (PMC8124660; doi:10.3390/ijerph18094703)
Supplement: Supplementary file 1 [file ijerph-18-04703-s001.zip › New folder/Supplementary/ijerph-1166592-Supplementary 2.pdf]

Table S2. ASHR values (min, max, mean) per 100,000 inhabitants.

Year	All	Youth	Adults	Elderly
2014	Min = 256.0<br>Max = 846.7<br>x = 480.5	Min = 29.4<br>Max = 827.2<br>x = 268.6	Min = 147.3<br>Max = 571.1<br>x = 229.1	Min = 1269.9<br>Max = 4279.9<br>x = 2380.4
2015	Min = 306.3<br>Max = 1109.2<br>x = 490.8	Min = 46.6<br>Max = 997.6<br>x = 262.8	Min = 123.5<br>Max = 539.6<br>x = 223.9	Min = 1281.4<br>Max = 4886.4<br>x = 2511.29
2016	Min = 280.2<br>Max =8 21.3<br>x = 483.5	Min = 70.6<br>Max = 795.0<br>x = 242.4	Min = 99.5<br>Max = 446.8<br>x = 237.2	Min = 1323.3<br>Max = 5159.9<br>x = 2449.7
2017	Min = 270.0<br>Max = 879.3<br>x = 435.5	Min=26.2<br>Max=495.2<br>x = 208.6	Min = 119.6<br>Max = 346.1<br>x = 202.3	Min = 986.4<br>Max = 6230.0<br>x = 2301.5

Table S3. Moran index values.

Year	All	Youth	Adults	Elderly
2014	0.595	0.594	0.439	0.561
2015	0.522	0.648	0.351	0.561
2016	0.627	0.642	0.480	0.538
2017	0.526	0.550	0.488	0.519
