# Supplementary figures and images for: Spatial Patterns in Hospital-Acquired Infections in Portugal (2014–2017)
Source: Int J Environ Res Public Health. 2021 Apr 28;18(9):4703. doi: 10.3390/ijerph18094703 (PMC8124660; doi:10.3390/ijerph18094703)

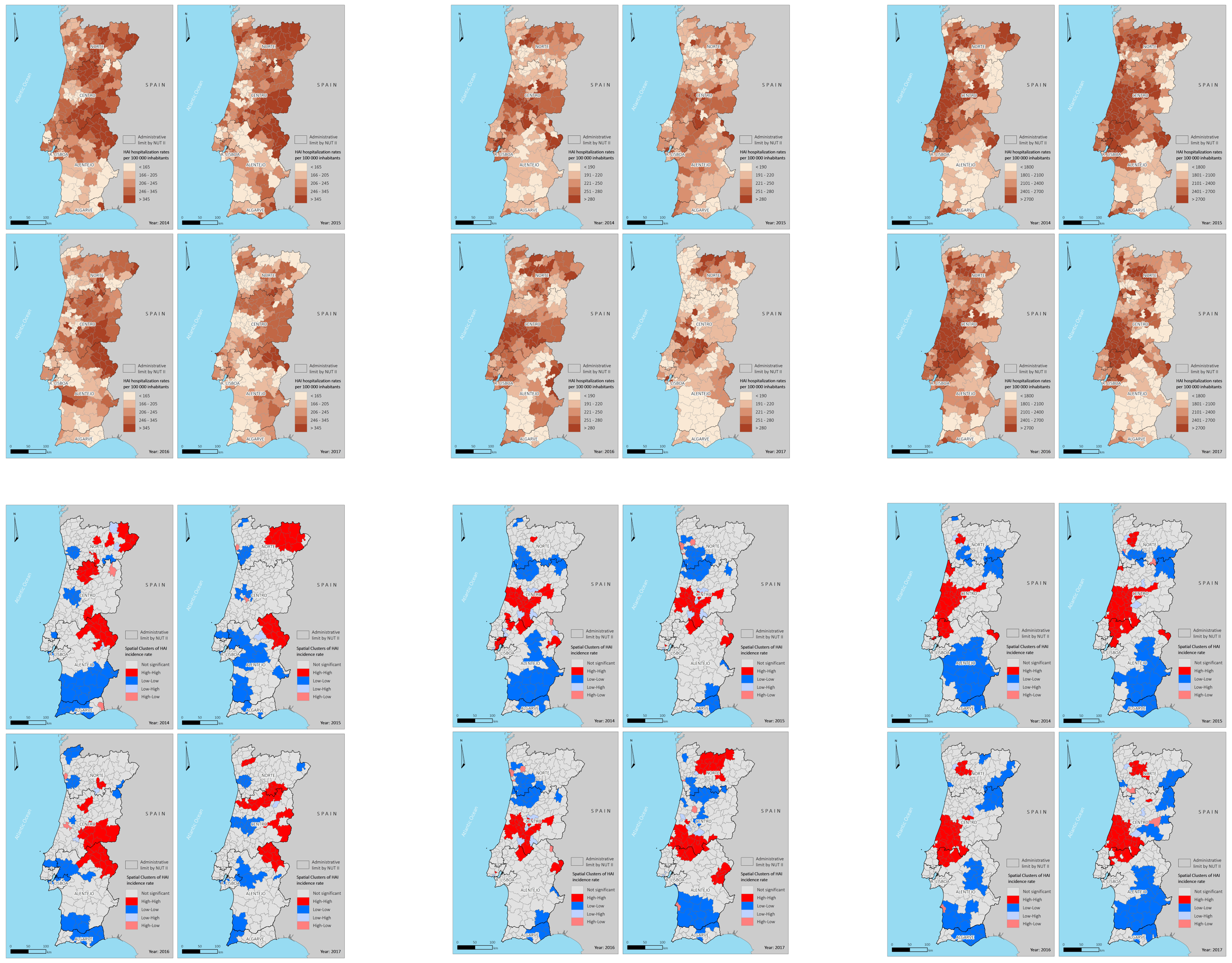

Supplement: Supplementary file 1 [file ijerph-18-04703-s001.zip › New folder/FIGURES/FigureS1.png]

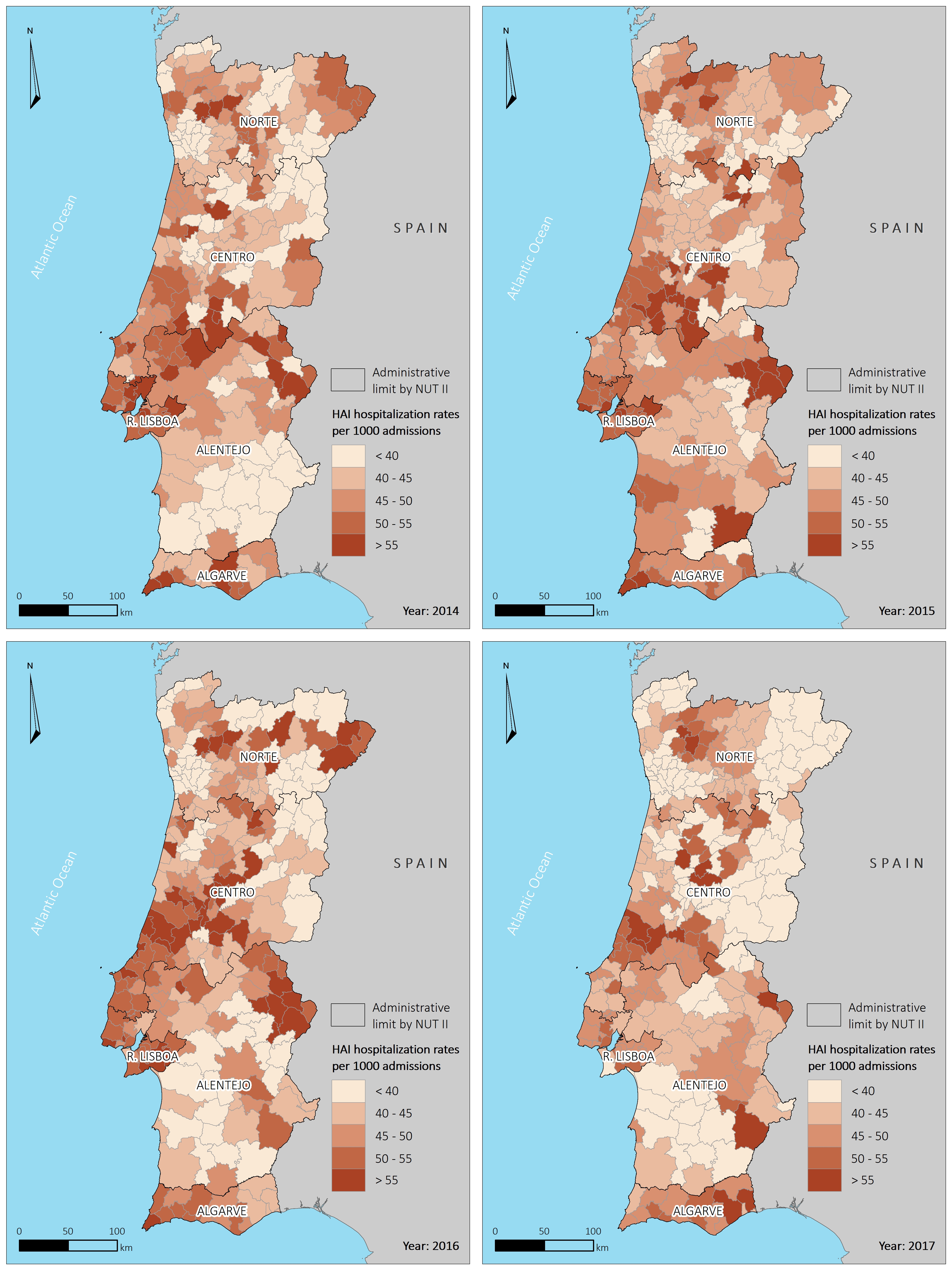

Supplement: Supplementary file 1 [file ijerph-18-04703-s001.zip › New folder/FIGURES/FigureS2.jpg]

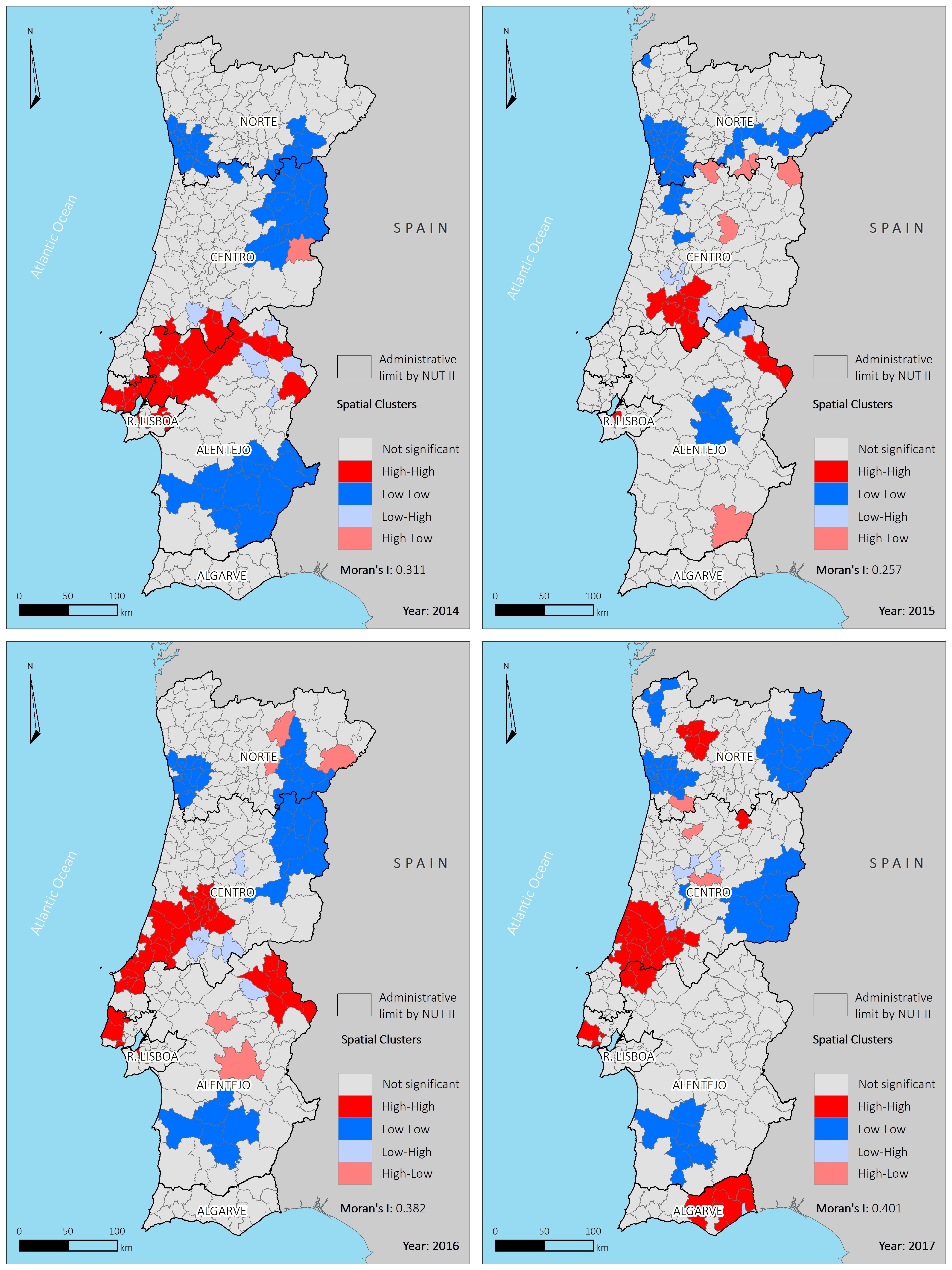

Supplement: Supplementary file 1 [file ijerph-18-04703-s001.zip › New folder/FIGURES/FigureS3.jpg]

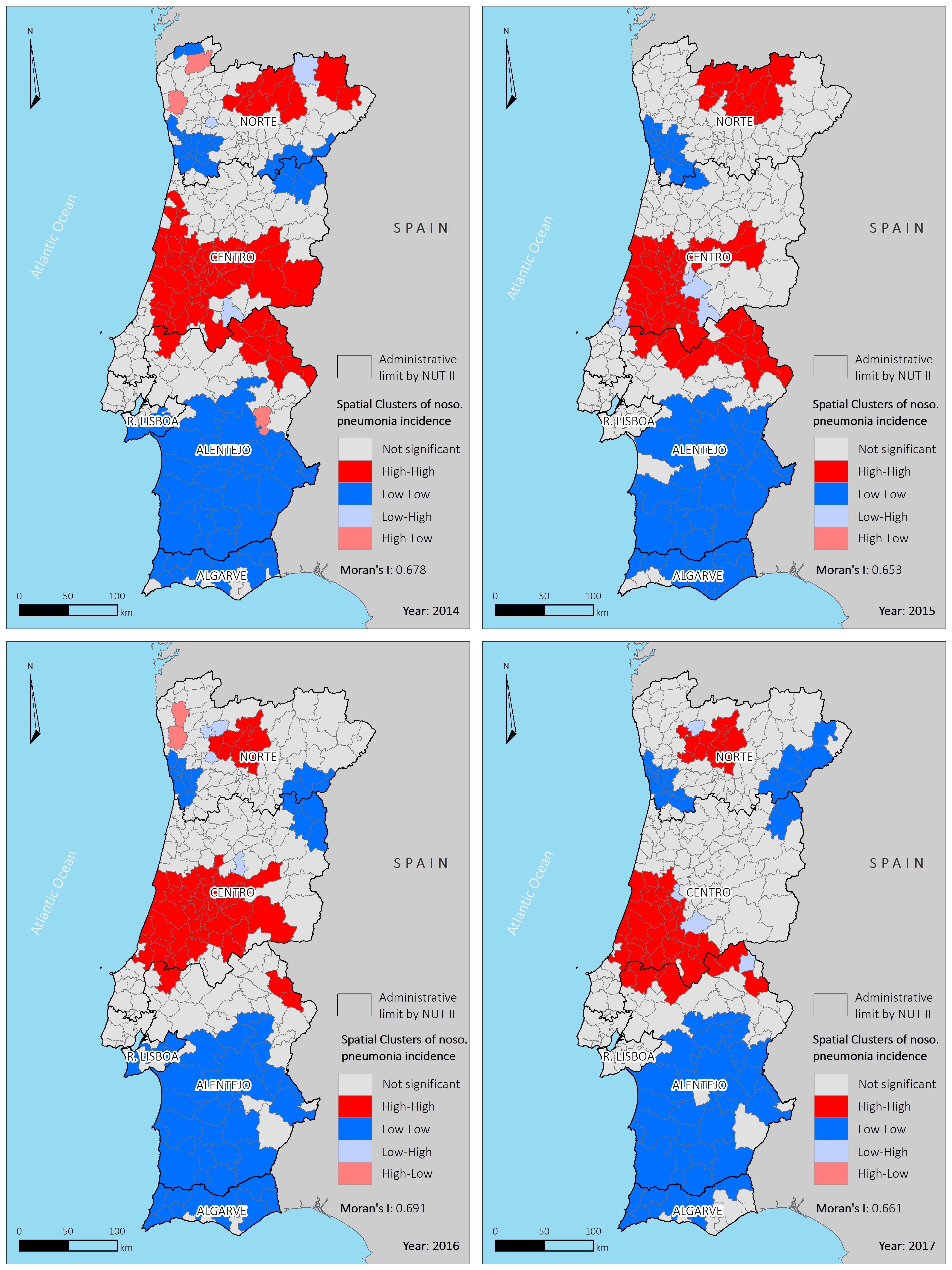

Supplement: Supplementary file 1 [file ijerph-18-04703-s001.zip › New folder/FIGURES/FigureS4.jpg]

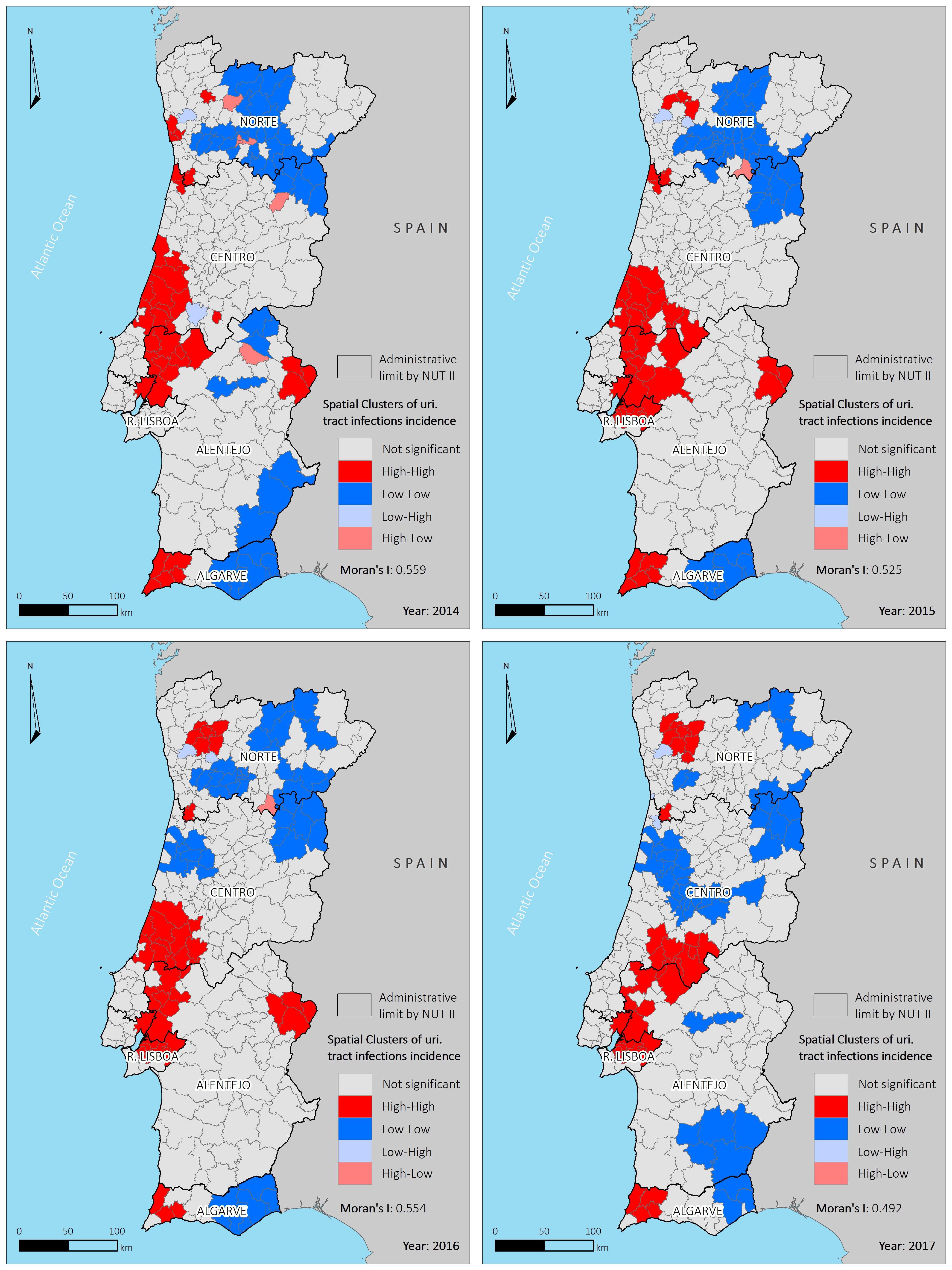

Supplement: Supplementary file 1 [file ijerph-18-04703-s001.zip › New folder/FIGURES/FigureS5.jpg]
